# Adherence to the World Cancer Research Fund/American Institute for Cancer Research recommendations for cancer prevention in adolescent and young adult (AYA) cancer survivors: results from the SURVAYA study

**DOI:** 10.1007/s11764-023-01529-4

**Published:** 2024-01-15

**Authors:** Costanza Gavioli, Carla Vlooswijk, Silvie H. M. Janssen, Suzanne E. J. Kaal, J. Martijn Kerst, Jacqueline M. Tromp, Monique E. M. M. Bos, Tom van der Hulle, Winette T. A. van der Graaf, Roy I. Lalisang, Janine Nuver, Rhodé M. Bijlsma, Mathilde C. M. Kouwenhoven, Olga Husson, Sandra Beijer

**Affiliations:** 1 Department of Research and Development, Netherlands Comprehensive Cancer Organisation, 3511 DT Utrecht, The Netherlands; 2 Department of Psychosocial Research and Epidemiology, Netherlands Cancer Institute, 1066 CX Amsterdam, The Netherlands; 3 Department of Medical Oncology, Netherlands Cancer Institute-Antoni Van Leeuwenhoek, 1066 CX Amsterdam, The Netherlands; 4 Department of Medical Oncology, Radboud University Medical Center, 6525 GA Nijmegen, The Netherlands; 5 Department of Medical Oncology, Amsterdam University Medical Center, 1105 AZ Amsterdam, The Netherlands; 6 Department of Medical Oncology, Erasmus MC Cancer Institute, 3015 GD Rotterdam, The Netherlands; 7 Department of Medical Oncology, Leiden University Medical Center, 2333 ZA Leiden, The Netherlands; 8 Department of Internal Medicine, GROW-School of Oncology and Reproduction, Maastricht UMC+ Comprehensive Cancer Center, 6229 HX Maastricht, The Netherlands; 9 Department of Medical Oncology, University Medical Center Groningen, 9713 GZ Groningen, The Netherlands; 10 Department of Medical Oncology, University Medical Center Utrecht, 3584 CX Utrecht, The Netherlands; 11 Department of Neurology, Amsterdam UMC, Amsterdam University Medical Centers, Location VUmc, 1081 HV Amsterdam, The Netherlands; 12 Department of Surgical Oncology, Erasmus MC Cancer Institute, Erasmus University Medical Center, 3015 GD Rotterdam, The Netherlands

**Keywords:** Adolescents and young adults with cancer (AYAs), Survivors, WCRF/AICR recommendations, Health behavior, Determinants

## Abstract

**Purpose:**

For adolescent and young adult (AYA) cancer survivors with a good prognosis, having a healthy lifestyle prevents morbidity and mortality after treatment. The aim of this study was to investigate the prevalence of (un)healthy lifestyle behaviors and related determinants in AYA cancer survivors.

**Methods:**

A population-based, cross-sectional study was performed among long-term (5–20 years) AYA cancer survivors (18–39 years old at diagnosis) registered within the Netherlands Cancer Registry. Self-reported questionnaires data about health behaviors were used to calculate the 2018 World Cancer Research Fund/American Institute for Cancer Research (WCRF/AICR) adherence score. Associations between the score and clinical/sociodemographic determinants of (un)healthy behaviors were investigated using logistic regression models.

**Results:**

The mean WCRF/AICR score was low to moderate, 3.8 ± 1.2 (0.5–7.0) (*n* = 3668). Sixty-one percent adhered to “limit the consumption of sugar sweetened drinks,” 28% to “be a healthy weight,” 25% to “fruit and vegetable consumption,” and 31% to “limit alcohol consumption.” Moderate and high adherence were associated with being a woman (OR_moderate_ = 1.46, 95% CI = 1.14–1.85, and OR_high_ = 1.87, 95% CI = 1.46–2.4) and highly educated (OR_moderate_ = 1.54, 95% CI = 1.30–1.83, and OR_high_ = 1.87, 95% CI = 1.46–2.4). Low adherence was associated with smoking (OR_moderate_ = 0.68, 95% CI = 0.50–0.92, and OR_high_ = 0.30, 95% CI = 0.21–0.44) and diagnosis of germ cell tumor (OR_moderate_ = 0.58, 95% CI = 0.39–0.86, and OR_high_ = 0.45, 95% CI = 0.30–0.69).

**Conclusions:**

Adherence to the 2018 WCRF/AICR lifestyle recommendations was low to moderate, especially regarding body weight, fruit, vegetables, and alcohol consumption. Men, current smokers, lower-educated participants, and/or those diagnosed with germ cell tumors were less likely to have a healthy lifestyle.

**Implications for Cancer Survivors:**

Health-promotion programs (e.g., age-specific tools) are needed, focusing on high-risk groups.

**Supplementary Information:**

The online version contains supplementary material available at 10.1007/s11764-023-01529-4.

## Introduction

Between pediatric and adult oncology, adolescents and young adults (AYAs) are a distinct, underserved, and understudied group in cancer care worldwide [1–3]. In the Netherlands, AYAs are defined as patients who are between 18 and 39 years at cancer diagnosis. The lower age is based on the Dutch health care system’s clear distinction between pediatric oncology (0 to 18 years) and adult oncology (18 years and older), while the upper age is based on cancer epidemiology [3]. No internationally agreed age definition of AYAs for cancer has been purposed [4].

Although rare, each year, an estimated 66.000 AYAs will develop cancer in Europe alone, representing 4% of all invasive cancer diagnoses [5]. Moreover, cancer is responsible for approximately 25% of all deaths at AYA age, making it the leading cause of disease-related death in this population in high-income countries all over the world. Improvement in the survival rate of several cancer types among AYAs is encouraging and survival now exceeds 80% at 5-years of follow-up [6, 7]. Nowadays, AYAs with a good prognosis can have a life expectancy of 50–60 years after cancer diagnosis, but the costs of survival are high due to long-term side effects and the risk of developing a new malignancy that is two to six times higher than in the general population [8].

Therefore, AYAs are advised to adhere to the World Cancer Research Fund/American Institute for Cancer Research (WCRF/AICR) recommendations for the prevention of cancer, developed both for the general population and for cancer survivors [9]. These recommendations are to maintain a healthy weight, be physically active, eat an optimal amount of fruit and vegetables, and limit the consumption of red and processed meat, fast foods, alcohol, and sweetened drinks. In addition to the above recommendations about physical activity and nutrition, the WCRF/AICR also suggests not smoking or limiting exposure to other tobacco products and excess sun as important behaviors to reduce cancer risk. Studies on cancer survivors outside of the AYA population already show that adherence to the WCRF/AICR recommendations is related to a lower risk of both overall and cancer-specific mortality [10]. For example, for pancreatic and colorectal cancer survivors as a whole, better adherence is associated with better physical, cognitive, and social functioning, a higher global health status and less fatigue [11–13].

However, little is known about adherence to a healthy lifestyle as a whole among AYAs, as previous research mostly focuses on adherence to single recommendations. As reported in a review by Carretier et al. [14], physical activity and fruit/vegetable consumption do not meet the recommendations both in AYA cancer survivors and in the general young adult population. Although AYA survivors seem to have a better knowledge of healthy lifestyle choices, they do not engage in healthier behaviors more than the general population of the same age, particularly in regard to tobacco smoking, maintenance of a balanced/healthy diet, and physical activity [14–16].

The determinants associated with an (un)healthy lifestyle among AYA cancer survivors have not been systematically addressed, although some factors have been identified. Studies among the general population and also among AYA cancer survivors show that females have a healthier lifestyle compared to males [15]. Female AYA survivors are more likely to smoke compared to AYA females in the general population, while male AYA survivors are less likely to smoke but more likely to drink alcohol compared to AYA males in the general population [16]. In both male and female childhood and adolescent cancer survivors, binge drinking was associated with a lower education level and a lower level of satisfaction about life. A binge drinker was defined as a respondent who reported having, on average, five or more alcoholic drinks on days that they drank [17]. Moreover, both male and female AYA survivors with low social and emotional support eat fewer fruit and vegetables, and female AYA survivors with low social and emotional support were less likely to be physically active [16]. On the contrary, AYA survivors receiving high social and emotional support and survivors within the first 10 years post-diagnosis were less likely to smoke.

Given that many AYAs have a long life ahead of them, having a healthy lifestyle as a whole is very important. Although several determinants have been found related to a single lifestyle behavior, it is unknown which determinants are associated with lifestyle as a whole. Moreover, studies on AYA cancer survivors often have a limited sample size and do not focus on long-term survivors. Therefore, the aim of this study is to investigate the level of adherence to WCRF/AICR recommendations (alcohol consumption, physical activity, nutrition, BMI, waist circumference) among a population-based sample of AYA cancer survivors and the determinants associated with an (un)healthy lifestyle. Understanding these determinants can help identify target groups who need more attention and which modifiable determinants are targets for interventions in order to enhance the adherence to a healthy lifestyle.

## Methods

### Design of the study

Data of the SURVAYA study were used. The SURVAYA study (health-related quality of life and late effects among SURVivors of cancer in Adolescence and Young Adulthood) is a retrospective, observational population-based cohort study, which was conducted among AYA long-term cancer survivors (5–20 years after initial diagnosis). They were selected from the Netherlands Cancer Registry (NCR), a population-based registry maintained by the Netherlands Comprehensive Cancer Organisation (IKNL). The complete overview of the SURVAYA study participants is provided in the paper by Vlooswijk et al. [18]. The SURVAYA study was conducted according to the Declaration of Helsinki guidelines and approved by the NKI Institutional Review Board (IRB-IRBd18122) and registered within clinical trial registration (NCT05379387).

### Study population

Participants included in the study were all AYA cancer survivors diagnosed with a primary cancer diagnosis at the age of 18–39 years between 1999 and 2015. The SURVAYA study was conducted in the Netherlands Cancer Institute and all University Medical Centers in the Netherlands.

### Data collection

Data collection was conducted between May 2019 and June 2021 within PROFILES (Patient Reported Outcomes Following Initial treatment and Long‐term Evaluation of Survivorship) [19], a data management system set up in 2009, linking these data with clinical data of the NCR.

All eligible AYA cancer survivors were informed of the study via a letter by their (ex-)attending medical doctor. The package also contained a secure link to log-in instructions, a web‐based informed consent form, and an online questionnaire. Participants were also given the option to request a paper version of the questionnaire that they could return by post. A reminder was sent within a timeframe of 2–7 months (it was not possible to send the reminders in the hospitals due to COVID-19) to non-responders in the same way as the first invitation. Patients were assured that non-participation had no consequences for their treatment or follow-up care. Details of the invitation procedures are described elsewhere [18].

### Measures

#### Demographic and clinical data

Sociodemographic (sex and date of birth) and clinical characteristics (tumor type, cancer stage, primary treatment, and date of diagnosis) were available from the NCR. Tumor type was classified according to the third International Classification of Diseases for Oncology (ICDO-3) [20]. For tumor type, we expected differences in adherence between the groups because it was hypothesized that tumor type could determine differences in lifestyle. We combined similar tumor types if we did not expected differences in lifestyle (See Table 2). Cancer stage was classified according to TNM or Ann Arbor Code (Hodgkin lymphoma and non-Hodgkin lymphoma) [21]. TNM 5 was used for patients diagnosed from 1999 to 2002, TNM 6 for patients diagnosed from 2003 to 2009, and TNM 7 was used for patients diagnosed from 2010 to 2015. For chronic lymphocytic leukemia, multiple myeloma, stage was not determined nor registered.

#### Self-reported data

Race/ethnicity (white/Caucasian or other), living situation (alone/with partner, with partner and children, with children only and “other”), work situation (employed/not employed), and education level (lower and higher) were self-reported. For living situation, more specific groups than alone/not alone were created to better study the different influence of every situation on lifestyle. Moreover, the “other” group defined participants living with parents or roommates. The low education level defined participants who did secondary school or less, while we identified those who did college or university as highly educated. Information about lifestyle was assessed through a self-reported questionnaire (Supplementary file 1). We assessed physical activity (PA) with questions derived from the validated European Prospective Investigation into Cancer (EPIC) Physical Activity Questionnaire [22]. Participants were asked how much time they spend on the following activities (average number of hours per week (hrs/wk), in summer and winter separately): walking, cycling, gardening, housekeeping, and sports. To include an estimate of intensity, metabolic equivalent intensity values (MET) were assigned to each activity, according to the compendium of physical activities [23, 24]. After selecting moderate and vigorous physical activity (MVPA, MET ≥ 3), we computed the mean number of hrs/wk of MVPA. Total PA was calculated by summing hrs/wk of all activities excluding household activities (MET = 3.5) that were not considered to be MVPA in accordance with previous research [24, 25]. Because about 98% of AYA survivors adhered to the Dutch physical activity guideline of 2.5 h of MVPA per week, it was not possible to use this as a cutoff point to categorize survivors in little or very active. Therefore, we categorized survivors in tertiles of minutes per week spent on MVPA, while survivors with missing values for MVPA (*n* = 225) were excluded. Height and weight were self-reported without any additional instructions about how to measure them. Survivors were categorized by their BMI status into underweight (BMI < 18.5 kg/m^2^), healthy weight (18.5– < 24.9 kg/m^2^), overweight (BMI 25– < 30 kg/m^2^), and obese (BMI ≥ 30 kg/m^2^). The questionnaire also included instructions and a tape measure so that patients could assess their own waist circumference. Cutoffs were < 94 cm, between 94 and 102 cm and ≥ 102 cm for men and < 80 cm, between 80 and 88 cm and ≥ 88 cm for women, based on the cutoff points by the 2018 WCRF/AICR Recommendation, Centers for Disease Control and Prevention [26], and the National Heart, Lung, and Blood Institute guidelines [27].

Smoking and alcohol use were assessed using self-developed questions about smoking behavior and alcohol (ab)use (do you smoke/drink alcohol? No/No but I used to/Yes). For alcohol intake, we asked participants to indicate the number of glasses consumed per week. Drug (ab)use was also assessed (have you ever used drugs? Yes/No), and then drug users were classified as never users (defined as those who reported having never used drugs), occasional drug users (defined as those who reported using drugs yearly, or a few times a year), and regular drug users (defined as those who reported using drugs monthly, weekly, or daily).

Dietary habits were assessed through a 10-item self-administered questionnaire. The food groups included in the questionnaire were “Vegetables,” “Fruit,” “Cookies, cakes, chips,” “Red meat,” “Processed meat,” “Sweetened drinks,” and “Fast foods.” In the questionnaire, more explanations were given regarding “red and processed meat” and “sugar sweetened drinks” (Supplementary file 1). Frequency of food consumption was measured in eight categories of frequency (ranging from never or almost never to > 7 times a week). For the groups “Vegetables,” “Fruit,” and “Sweetened drinks,” we also asked indication of the number of portions consumed, through answers ranging from 1 to 7 portions a day and “not applicable.” A portion across the respective categories was considered to be a spoon of vegetables equal to 50 g, a piece of fruit equal to 125 g, and a glass of sweetened drinks equal to 250 ml. For these items, we transposed daily food consumption to grams and estimated by multiplying the portion size by the consumption frequency for each food item.

#### WCRF/AICR Recommendations Adherence Score

The adherence to the 2018 WCRF/AICR cancer prevention recommendations was quantified using the standardized score as developed by Shams-White et al. [28], and an indicator for overall lifestyle was used [9]. We operationalized seven recommendations, focusing on BMI and waist circumference, physical activity, fruit and vegetables, ultra-processed foods, red and processed meat, sugary drinks, and alcohol. An overview of the applied WCRF/AICR lifestyle (sub)recommendations, as well as the operationalization of the recommendations, and scoring thereof are shown in Table 1. For the classification of ultra-processed foods, we included foods categorized in the 4th group of the NOVA, a classification system that groups all foods according to the nature, extent, and purpose of the industrial processes they undergo. These involve physical, biological, and chemical techniques used after foods are separated from nature, and before they are consumed or else made into dishes and meals. We included in this category the food groups “Cookies, cakes, chips” and “Fast foods” [29, 30]. We did not include food groups overlapping with other score components such as processed meats and sugar-sweetened beverages.

**Table 1:** Operationalization of lifestyle recommendations for the prevention of cancer by the World Cancer Research Fund and American Institute for Cancer Research (WCRF/AICR)

	2018 WCRF/AICR cancer prevention recommendations	Study data	Operationalization of recommendation	Deviations from 2018 WCRF/AICR official score	Lifestyle score
1	Be a healthy weight	BMI (kg/m^2^)	18.5–24.9		0.5
			25–29.9		0.25
			< 18.5 or ≥ 30		0
		Waist circumference (cm)	Men: < 94		0.5
			Women: < 80		
			Men: 94- < 102		0.25
			Women: 80- < 88		
			Men: ≥ 102		0
			Women: ≥ 88		
2	Be physically active	Total moderate-vigorous physical activity (min/week)	3rd tertile (> 825)	≥ 150	1
			2nd tertile (435 < / ≤ 825)	75– < 150	0.5
			1st tertile (≤ 435)	< 75	0
3	Eat a diet rich in whole grains, vegetables, fruit, and beans	Fruit and vegetables intake (g/day)	≥ 400		1
			200- < 400		0.5
			< 200		0
4	Limit consumption of “fast foods” and other processed foods high in fat, starches, or sugars	Biscuits, cakes, pies, chips, and fast food consumption (times/week)	3rd tertile (0–2)		1
			2nd tertile (3–4)		0.5
			1st tertile (≥ 5)		0
5	Limit consumption of red and processed meat	Red and processed meat consumption (times/week)	3rd tertile (0–2)	Red meat < 500 g/day and processed meat < 21 g/day	1
			2nd tertile (3–5)	Red meat < 500 g/day and processed meat 21– < 100 g/day	0.5
			1st tertile (≥ 6)	Red meat > 500 g/day or processed meat ≥ 100 g/day	0
6	Limit consumption of sugar-sweetened drinks	Total sugar-sweetened drinks intake (g/day)	0		1
			> 0 ≤ 250		0.5
			> 250		0
7	Limit alcohol consumption	Total ethanol intake (g/day)	0 (men and women)		1
			Men: > 0– ≤ 28		0.5
			Women: > 0– ≤ 14		
			Men: > 28		0
			Women: > 14		

*BMI* body mass index, *cm* centimeters, *g* grams, *kcal* kilocalories, *kg* kilograms, *m* meters, *min* minutes

For each recommendation, we assigned a score of 1 (full adherence), 0.5 (partial adherence), or 0 (low adherence). For the recommendation “be a healthy weight,” the sum of the score for BMI and waist circumference was used. When only one parameter was available, the value was doubled to score (i.e., in both scenarios, this subcomponent’s total range will remain 0–1).

As recommended by Shams-White et al. [29], we made some adaptations to the score to assess adherence. The recommendation “eating a diet rich in whole grains, vegetables, fruits, and beans” is originally composed of two sub-recommendations. However, in our study, only fruit and vegetable intake could be scored (one sub-recommendation). Given that the total score per recommendation must be 1, the sub-recommendation’s score for fruit and vegetables was also doubled. Moreover, we used pre-defined cutoff criteria with the exception of physical activity and red and processed meat. As our study did not include adequate measures that align with the standardized scoring system, we used tertiles: moderate to vigorous physical activity: ≤ 435 min/week, 435–825 min/week, and > 825 min/week; red and processed meat: 0–2, 3–5, and ≥ 6 times a week. For ultra-processed foods, tertiles were 0–2, 3–4, and ≥ 5 times a week.

The final WCRF adherence score was obtained by summing up the scores of (sub)recommendations and ranged from 0 to 7, with higher scores indicating better adherence.

We categorized the population into three groups based on tertiles of WCRF/AICR adherence scores: group 1: score 0.5–3, group 2: score 3.25–4.25, and group 3: score 4.5–7.

#### Statistical analysis

During the data cleaning procedures, height values less than 100 cm and weight below 39 kg were marked as missing, since they were matched with other unrealistic weights or heights in the questionnaire. For waist circumference, any extreme value below 57 cm was set to missing, as were values with discrepancies with the reported weight and height. No upper limit was set as the highest values appeared consistent with the corresponding reported weights and heights.

Demographic and lifestyle characteristics were described of the total population and for the three WCRF/AICR adherence groups individually as frequencies with percentages for categorical variables and means with standard deviations for continuous variables. Chi-square (categorical variables) and one-way ANOVA (continuous variables) were used to test differences in baseline characteristics between the three WCRF/AICR adherence groups.

To assess the association between the WCRF adherence scores and several determinants, multinomial logistic regression models were used. We included adherence to the WCRF score (tertiles) as the dependent determinant and the variables gender, age at the time of questionnaire, years since diagnosis, ethnicity, education level, living situation, working situation, tumor stage, tumor type, type of treatment, smoking status, and use of drugs were separately included as independent variables in the univariate analyses. We expressed results as ORs and 95% Cis and included all determinants which were significantly associated with the WCRF adherence score (tertiles) in the univariate analyses in the multivariate multinomial model.

Statistical analyses were conducted using SAS software version 9.4. (SAS Institute, Cary, NC). A *p*-value < 0.05 was considered statistically significant.

## Results

### General characteristics of the study population

In total, 11,340 AYA survivors were invited to participate in the study of whom 4010 completed the questionnaire (36%). We excluded from the analysis AYA cancer survivors with missing data on the WCRF/AICR adherence (*n* = 342). Females, AYAs of non-Caucasian ethnicities, and those with lower educational levels were more likely to have missing data on the WRC/AICR adherence score (data not shown). In Table 2, demographic and lifestyle characteristics of the 3668 AYA cancer survivors with complete data are shown. Survivors were more often to be woman (60.7%), with a mean age ± SD at time of questionnaire of 44.5 ± 7.5 years, and had been diagnosed on average 12.4 ± 4.5 years before filling in the questionnaire.

**Table 2:** Baseline characteristics of Dutch AYA survivors

	All patients (*N* = 3668)	WCRF/AICR tertile 1* (*N* = 1107)	WCRF/AICR tertile 2* (*N* = 1313)	WCRF/AICR tertile 3* (*N* = 1248)	*p*-value
Gender, *n* (%)					< 0.0001
Women	2228 (60.7)	531 (48.0)	828 (63.1)	869 (69.6)	
Age at time of questionnaire					0.1719
Mean (SD)	44.5 (7.5)	44.2 (7.3)	44.7 (7.5)	44.6 (7.6)	
Years since diagnosis					0.6655
Mean (SD)	12.4 (4.5)	12.5 (4.4)	12.5 (4.5)	12.3 (4.6)	
Years since diagnosis, *n* (%)					0.3236
5–10 years	1256 (34.2)	358 (32.3)	458 (34.9)	440 (35.3)	
11–15 years	1278 (34.8)	412 (37.2)	440 (33.5)	426 (34.1)	
16–20 years	1134 (30.9)	337 (30.4)	415 (31.6)	382 (30.6)	
Ethnicity, *n* (%)					0.0686
White/Caucasian	3301 (90.1)	1006 (91.0)	1191 (90.8)	1104 (88.5)	
Other	362 (9.9)	99 (9.0)	120 (9.2)	143 (11.5)	
Education level, *n* (%)					< 0.0001
Low	1577 (43.1)	604 (54.7)	572 (43.6)	401 (32.2)	
High	2084 (56.9)	500 (45.3)	739 (56.4)	845 (67.8)	
Living situation, *n* (%)					< 0.0001
Alone	461 (12.6)	118 (10.7)	148 (11.3)	195 (15.7)	
With partner	1046 (28.6)	259 (23.4)	381 (29.1)	406 (32.6)	
With partner and children	1550 (42.3)	538 (48.7)	584 (44.6)	428 (34.4)	
With children only	481 (13.1)	151 (13.7)	158 (12.1)	172 (13.8)	
Other	122 (3.3)	39 (3.5)	39 (3.0)	44 (3.5)	
Work situation, *n* (%)					0.3891
Current worker	3086 (84.3)	932 (84.3)	1117 (85.2)	1037 (83.2)	
Tumor stage, *n* (%)					0.7637
I	1571 (48.5)	453 (47.0)	561 (48.4)	557 (50.0)	
II	968 (29.9)	290 (30.1)	357 (30.8)	321 (28.8)	
III	529 (16.3)	170 (17.7)	180 (15.5)	179 (16.1)	
IV	168 (5.2)	50 (5.2)	62 (5.3)	56 (5.0)	
Missing^1^	432	144	153	135	
Tumor type, *n* (%)					< 0.0001
Melanoma	259 (7.1)	64 (5.8)	96 (7.3)	99 (7.9)	
Breast	865 (23.6)	182 (16.4)	325 (24.8)	358 (28.7)	
Female genitalia	392 (10.7)	93 (8.4)	152 (11.6)	147 (11.8)	
Germ cell tumors	646 (17.6)	283 (25.6)	213 (16.2)	150 (12.0)	
Hematological malignancies^2^	681 (18.6)	205 (18.5)	247 (18.8)	229 (18.3)	
Thyroid gland	227 (6.2)	69 (6.2)	76 (5.8)	82 (6.6)	
Other^3^	598 (16.3)	211 (19.1)	204 (15.5)	183 (14.7)	
Type of treatment, *n* (%)					
Chemotherapy	2060 (56.2)	615 (55.7)	751 (57.2)	694 (55.7)	0.6502
Radiotherapy, *n* (%)	1750 (47.8)	495 (44.8)	642 (48.9)	613 (49.2)	0.0611
Hormonal therapy, *n* (%)	444 (12.1)	96 (8.7)	159 (12.1)	189 (15.2)	< 0.000
Targeted therapy, *n* (%)	281 (7.7)	72 (6.5)	105 (8.0)	104 (8.3)	0.2149
Surgery, *n* (%)	2860 (78.1)	863 (78.1)	1014 (77.3)	983 (78.8)	0.6408
Stem cell therapy, *n* (%)	134 (3.7)	46 (4.2)	49 (3.7)	39 (3.1)	0.4030^1^
Smoking status, *n* (%)					< 0.0001
Never	2091 (57.0)	580 (52.4)	740 (56.4)	771 (61.8)	
Former	1271 (34.7)	390 (35.3)	463 (35.3)	418 (33.5)	
Current	304 (8.3)	136 (12.3)	110 (8.4)	58 (4.7)	
Use of drugs, *n* (%)					0.0064
Never	2764 (75.4)	836 (75.6)	1017 (77.5)	911 (73.0)	
Occasionally	647 (17.6)	177 (16.0)	217 (16.5)	253 (20.3)	
Regularly	256 (7.0)	93 (8.4)	79 (6.0)	84 (6.7)	
Adherence to recommendations, *n* (%)					
Weight					< 0.0001
0	421 (11.5)	254 (22.9)	116 (8.8)	51 (4.1)	
0.25	750 (20.4)	275 (24.8)	354 (27.0)	121 (9.7)	
0.5	742 (20.2)	267 (24.1)	229 (17.4)	246 (19.7)	
0.75	743 (20.3)	135 (12.2)	346 (26.4)	262 (21.0)	
1	1012 (27.6)	176 (15.9)	268 (20.4)	568 (45.5)	
Physical activity					< 0.0001
0	1132 (30.9)	592 (53.5)	351 (26.7)	189 (15.1)	
0.5	1249 (34.1)	341 (30.8)	525 (40.0)	383 (30.7)	
1	1287 (35.1)	174 (15.7)	437 (33.3)	676 (54.2)	
Fruit and vegetables					< 0.0001
0	1037 (28.3)	588 (53.1)	316 (24.1)	133 (10.7)	
0.5	1713 (46.7)	469 (42.4)	759 (57.8)	485 (38.9)	
1	918 (25.0)	50 (4.5)	238 (18.1)	630 (50.5)	
Fast foods and other processed foods					< 0.0001
0	1426 (38.9)	733 (66.2)	503 (38.3)	190 (15.2)	
0.5	1081 (29.5)	294 (26.6)	459 (35.0)	328 (26.3)	
1	1161 (31.7)	80 (7.2)	351 (26.7)	730 (58.5)	
Red and processed meat					< 0.0001
0	1596 (43.5)	826 (74.6)	623 (47.4)	147 (11.8)	
0.5	1154 (31.5)	249 (22.5)	483 (36.8)	422 (33.8)	
1	918 (25.0)	32 (2.9)	207 (15.8)	679 (54.4)	
Sugar-sweetened drinks					< 0.0001
0	403 (11.0)	305 (27.6)	88 (6.7)	10 (0.8)	
0.5	1022 (27.8)	459 (41.5)	404 (30.8)	159 (12.7)	
1	2243 (61.1)	343 (31.0)	821 (62.5)	1079 (86.5)	
Alcohol consumption					< 0.0001
0	247 (6.7)	93 (8.4)	100 (7.6)	54 (4.3)	
0.5	2279 (61.8)	766 (69.2)	817 (62.2)	696 (55.8)	
1	1142 (31.4)	248 (22.4)	396 (30.2)	498 (39.9)	

Missing < 5% are not shown in the table. Missing: Ethnicity = 5; education level = 7; living situation = 8; work situation = 6; type of treatment = 4; smoking status = 2; use of drugs = 1.^2^ “Hematological malignancies” include both lymphoid and myeloid hematological malignancies.^3^Other include the following tumor types: “Head and neck,” “Colon and rectal,” “Digestive tract,” “Respiratory tract,” “Bone and soft tissue sarcoma,” “Male genitalia,” “Urinary tract,” “Other,” “Central nervous system”. *WCRF/AICR adherence score: tertile 1: score 0.5–3, tertile 2: score 3.25–4.25, tertile 3: score 4.5–7

### WCRF/AICR adherence score

The mean total WCRF/AICR adherence score was 3.8 ± 1.2 of a total of 7 points (range 0.5–7.0). Higher WCRF/AICR adherence scores were more common among women compared to men. The highest WCRF/AICR adherence scores were found among survivors who had a higher education level, were living alone, had never smoked, and used drugs occasionally and among those diagnosed with breast cancer or melanoma and treated with hormonal therapy (HT) (Table 2). The mean adherence score was 2.7 ± 1.0 of a total of 5 points (range 0–5) for the dietary recommendations. For the distribution of BMI, waist circumference and consumption of vegetables, fruit, sweetened drinks, and alcoholic drinks among participants of the study, see Fig. 1a-f (Supplementary file 2).

### Determinants of (un)healthy lifestyle

The results of the univariate and multivariate logistic regression analysis on the determinants of (un)healthy lifestyle are shown in Table 3.

**Table 3:** Associations between adherence to the World Cancer Research Fund/American Institute for Cancer Research (WCRF/AICR) lifestyle recommendations with baseline characteristics in AYA survivors

	WCRF adherence scores							
	Univariable				Multivariable			
	Moderate versus low adherence		High versus low adherence		Moderate versus low adherence		High versus low adherence	
	3–4.25 points		> 4.25 points		3–4.25 points		> 4.25 points	
	Odds ratio	(95% CI)	Odds ratio	(95% CI)	Odds ratio	(95% CI)	Odds ratio	(95% CI)
Gender (reference: men)	1		1		1		1	
Women	1.85	(1.57–2.18)	2.49	(2.10–2.94)	1.46	(1.14–1.85)	1.87	(1.46–2.4)
Age at questionnaire (continuous)	1.01	(1.00–1.02)	1.01	(1.00–1.02)				
Years since diagnosis (continuous)	1.00	(0.98–1.02)	0.99	(0.97–1.01)				
Ethnicity (reference: white/Caucasian)	1		1		1		1	
Other	1.02	(0.78–1.35)	1.32	(1.01–1.72)	1.05	(0.79–1.40)	1.41	(1.05–1.88)
Education level (reference: low-medium)	1		1		1		1	
High	1.56	(1.33–1.83)	2.55	(2.15–3.01)	1.54	(1.30–1.83)	2.48	(2.07–2.96)
Living situation (reference: alone)	1		1		1		1	
With partner	1.17	(0.88–1.57)	0.95	(0.72–1.25)	1.07	(0.79–1.44)	0.84	(0.63–1.13)
With partner and children	0.87	(0.66–1.13)	0.48	(0.37–0.63)	0.76	(0.57–1.00)	0.4	(0.3–0.52)
With children only	0.83	(0.60–1.16)	0.69	(0.50–0.95)	0.72	(0.51–1.00)	0.55	(0.4–0.78)
Other	0.80	(0.48–1.32)	0.68	(0.42–1.11)	0.84	(0.5–1.4)	0.72	(0.43–1.21)
Working situation (reference: not working)	1		1					
Working	1.07	(0.86–1.34)	0.92	(0.74–1.15)				
Smoking status (reference: never-smoker)	1		1		1		1	
Former smoker	0.93	(0.78–1.11)	0.81	(0.68–0.96)	0.94	(0.78–1.14)	0.75	(0.62–0.92)
Current smoker	0.63	(0.48–0.83)	0.32	(0.23–0.44)	0.68	(0.50–0.92)	0.30	(0.21–0.44)
Drugs use (reference: never)	1		1		1		1	
Occasionally	1.01	(0.81–1.25)	1.31	(1.06–1.62)	1.03	(0.82–1.30)	1.41	(1.12–1.79)
Regularly	0.70	(0.51–0.96)	0.83	(0.61–1.13)	0.91	(0.65–1.28)	1.45	(1.02–2.06)
Chemotherapy (reference: no)	1		1					
Yes	1.07	(0.91–1.25)	1.00	(0.85–1.18)				
Radiotherapy (reference: no)	1		1		1		1	
Yes	1.18	(1.01–1.39)	1.19	(1.01–1.40)	1.07	(0.89–1.29)	0.94	(0.77–1.15)
Hormonal therapy (reference: no)	1		1		1		1	
Yes	1.45	(1.11–1.89)	1.88	(1.45–2.44)	0.85	(0.59–1.22)	0.96	(0.67–1.34)
Targeted therapy (reference: no)	1		1					
Yes	1.25	(0.91–1.70)	1.31	(0.96–1.78)				
Surgery (reference: no)	1		1					
Yes	0.95	(0.79–1.16)	1.04	(0.86–1.27)				
Stem cells therapy (reference: no)	1		1					
Yes	0.89	(0.59–1.35)	0.74	(0.48–1.15)				
Tumor stage (reference: I) II	1 0.99	(0.82–1.21)	1 0.90	(0.74–1.10)				
III	0.86	(0.67–1.09)	0.86	(0.67–1.09)				
IV	1.00	(0.68–1.48)	0.91	(0.61–1.36)				
Tumor type (reference: breast)	1		1		1		1	
Female genitalia	0.92	(0.67–1.26)	0.80	(0.59–1.10)	0.88	(0.60–1.29)	0.81	(0.55–1.19)
Germ cell tumors	0.42	(0.33–0.54)	0.27	(0.21–0.35)	0.58	(0.39–0.86)	0.45	(0.30–0.69)
Hematological malignancies	0.68	(0.52–0.87)	0.57	(0.44–0.74)	0.79	(0.55–1.12)	0.72	(0.50–1.03)
Melanoma	0.84	(0.58–1.21)	0.79	(0.55–1.13)	0.91	(0.59–1.43)	0.89	(0.56–1.40)
Thyroid gland	0.62	(0.43–0.90)	0.60	(0.42–0.87)	0.64	(0.42–0.98)	0.72	(0.47–1.11)
Other	0.54	(0.42–0.71)	0.44	(0.34–0.58)	0.63	(0.44–0.90)	0.58	(0.40–0.83)

Multivariable multinomial logistic regression analysis showed that being a woman and highly educated were also independently associated with a moderate and high adherence. Current smokers compared to never-smokers were less likely to have a moderate or high adherence. AYAs diagnosed with germ cell tumor or “other” tumors were less likely to have a moderate or high adherence. Ethnicities different from white/Caucasian and participants using drugs occasionally or regularly compared to never-users were more likely to have a high adherence to the WCRF recommendations. Participants diagnosed with thyroid cancer were less likely to have a moderate adherence when compared to those diagnosed with breast cancer. Participants living with partner and child(ren) and only with child(ren) were less likely to have a high adherence. Age at the time of the questionnaire, years since diagnosis, working situation, stage of the disease, and other types of treatment were not associated with the level of adherence.

## Discussion

In this population-based cross-sectional study, we found that overall adherence to the 2018 WCRF/AICR recommendations in AYA cancer survivors was low to moderate, particularly for the maintenance of a healthy weight and the consumption of fruit, vegetables, and alcohol. Being a woman and having a high education level were significantly associated with a higher WCRF/AICR score, while smoking and diagnosis of germ cell tumor were associated with a lower WCRF/AICR score.

### WCRF/AICR score adherence among cancer survivors, AYA cancer survivors, and general population

As far as we know, no previous studies have calculated the adherence to the WCRF/AICR score in AYA cancer survivors specifically. The generally low to moderate overall adherence to the WCRF/AICR recommendations in our study is in line with the findings in other populations of cancer survivors. A Dutch study in colorectal cancer (CRC) survivors reported a mean score of 4.8 out of a maximum of 8 points, while another study in elderly female cancer survivors showed an average adherence of 4.0 of 7 points. However, scoring of adherence to the guidelines was not completely comparable because of the differences in the number and types of recommendations and their operationalization, since many of these studies were based on the 2007 WCRF/AICR score [11, 12, 31].

The level of adherence to some of the individual lifestyle behaviors was very variable. Prevalence of smoking was in line with our findings among AYAs [14, 15, 32] but higher among the general population [33]. Fruit and vegetables consumption was in line with our findings among AYAs [34]. Considering body weight, the number of AYAs classified as overweight or obese, it was comparable with other studies among AYAs from Canada and Australia, which found a prevalence between 47 and 56% (Fig. 1a, Supplementary file) [34, 35], and was also consistent with the finding in a study among Dutch adult cancer survivors [12]. In the general population, the prevalence of body weights classified as overweight (50% vs 32.4%) and obese (14.3% vs 11.7%) was higher compared to our findings [33]. These results could suggest that AYA cancer survivors do not have more difficulties in maintaining a healthy BMI than the general population. This is different from childhood cancer survivors who often struggle to maintain a healthy weight [36]. Alcoholic consumption was higher in our cohort compared to other studies in AYA cancer survivors [15, 35] and among the general population [33].

It is very hard to compare the level of physical activity with other studies because in our cohort, physical activity is probably overestimated due to the way of questioning. In fact, 98% of participants adhered to the recommendation of 150 min/week of moderate or intense PA. The systematic review and meta-analysis from Tollosa et al. [32] about adherence to health behaviors in cancer survivors (mean age = 56.9 years) showed different levels of adherence to MVPA, ranging from 12 to 78%. Moreover, in other studies among AYA cancer survivors (mean age 40 years), adherence was 31% [37]. Our results differ in comparison to the data of the general population, which report that only 50.8% of Dutch adults (age range 18–54 years) meets the physical activity level recommended by the Dutch Standards for Healthy Exercise (NNGB) [38, 39]. However, this is not a scoring system, but a guideline established in 1998 and based on consensus of national experts and international publications addressing young people, adults, and older adults. The differences between results from the literature and our results may partly be explained by methodological reasons, especially by the use of other methods to measure dietary intake and physical activity. Moreover, body height and weight were assessed through self-reported questionnaires in some studies, while in other studies, these were obtained from each survivor’s chart. In addition, different definitions of adherence could explain few differences. Our study was the first one that used the score based on the 2018 WCRF/AICR cancer prevention recommendations, while the others were based on the 2007 WCRF/AICR cancer prevention recommendations [40], and, moreover, no studies among adherence of AYA cancer survivors were conducted. In the most recent version, the total score ranges from 0 to 7 points for men and 0 to 8 for women, while the previous one from 0 to 6 for men and 0 to 7 for women (because of the recommendation about breastfeeding). This is due to the fact that the recommendations about the consumption of energy-dense food, sugary drinks, and fast foods changed significantly between 2007 and 2018 recommendations, and was split in two recommendations in the latter. In addition, the new definition of “be a healthy weight” is made of two sub-recommendations about BMI and waist circumference, while the previous one only considered BMI. The same is the case for alcohol consumption: in the 2018 WCRF/AICR score, the maximum score is given to participants who do not consume alcohol at all, while in studies that used the 2007 recommendations or other guidelines, a minimum consumption was allowed, so the cutoffs were higher.

### Determinants of WCRF/AICR score adherence among cancer survivors, AYA cancer survivors, and the general population

We found that gender (women), high education level, and never smoking were determinants associated with the healthiest behaviors, and this was in line with results of other studies [11, 14, 41]. Women were more likely to have a healthier lifestyle, and the same result was found both in Dutch CRC survivors, AYAs, childhood cancer survivors, and in the general population [15, 42, 43]. Women in general have more health-conscious behaviors and also greater consumption of fruit and vegetables, whole grains, better adherence to a low fat diet, and consume less alcohol. In a study conducted among healthy premenopausal women aged 20–50 years, it seems that women are more self-determined in a diversity of lifestyle domains such as physical activity and sport, education and well-being, and save for more nutritional knowledge [44]. A systematic review of the literature [45] showed that a higher education level was the psychosocial determinant most associated with favorable lifestyle changes after a cancer diagnosis, and to a higher adherence to the recommendations.

Our findings about living situation, years since diagnosis, ethnicities other than white/Caucasian, and drug use as determinants of an (un)healthy lifestyle are not consistent with the results in the literature. Participants living with partner and children or only with children were more likely to have an unhealthy lifestyle when compared to those living alone, while other studies showed a better adherence among cancer survivors with higher social network diversity such as the presence of a partner and living relatives [46]. Moreover, previous studies among AYAs showed that more recent survivors (5–10 years) generally have a healthier lifestyle than long-term survivors (> 10 years), while this was not evident in our cohort [16]. Rabin et al. [47] observed that cancer survivors in the older spectrum of the young adult age range tended to have an unhealthier lifestyle. They were less physically active and had higher prevalence of smoking and smoked heavily, though they were less likely to drink heavily. In our cohort, ethnicities other than white/Caucasian have better adherence to the WCRF/AICR recommendations, while in the general population in the Netherlands, a higher adherence to a “healthy cluster” was found in white Dutch people compared to ethnically Turkish, Moroccans, and Surinamese/Antillean people in the Netherlands [48]. It has also been demonstrated that immigrants in general have higher prevalence of overweight and obesity, smoking, eating takeout and delivery food, and lower physical activity levels when compared to Dutch responders [49]. However, we assume that the differences in our results could be due to the small sample size of the “other” group that included ethnicities other than white/Caucasian, with only 362 participants. Statistics Netherlands [33] shows that, in 2022, 25.2% of people in the Netherlands had a migration background (defined as a person of whom at least one parent is born outside the Netherlands), while in our cohort, only 9.1% of people involved belong to ethnicities different than white/Caucasian. As shown in an American study among patients suffering from chronic kidney disease, this difference could be explained to the likelihood that ethnicities other than white/Caucasian are more likely to have a low socio-economic and health literacy. Consequently, they might be less inclined to participate in clinical studies [50].

The 9.1% of people with ethnicities different than white/Caucasian in our study might have contributed to a slight overrepresentation of higher educated immigrants, which could explain better lifestyle habits [49].

Occasional and regular drug users were more likely to adhere better to a healthy lifestyle. Studies about cannabis use in (childhood) cancer survivors found that users were more educated, and infrequent users were more likely to do physical activity compared to non-users [51]. Moreover, among a general population in the Netherlands, we know that most drug users are highly educated, men, white/Caucasian, and with a regular nightlife [52]. Therefore, we can expect that in our cohort, AYA cancer survivors with a higher education level are more likely to use drugs, and this could explain the relation with a higher adherence to the recommendations.

In our cohort, patients with breast cancer had the healthiest lifestyle compared to other tumor types. This result could be influenced by the fact that this type of cancer mostly affects women and may get more attention in general with lifestyle advice compared to other cancers. Although found to be not significant, patients diagnosed with female genitalia cancers had the best adherence after breast cancer, while those with germ cell tumors, a typical male tumor type, had the worst adherence. This could be explained by the fact that, as reported by Gietema et al. [53], patients treated with chemotherapy for testicular cancer have an unexpected increase in BMI 4 to 6 years after treatment, not only because of (thyroid) hormonal change but also for a reported increased caloric intake after stressful events in life and a decreased physical activity. This could possibly explain lower WCRF/AICR scores in these patients. In the end, this study did not ask for the development of second malignancies among participants, but it is well established that obesity, unhealthy diets, alcohol use, and smoking play a significant role in the development of a second malignancy [54].

### Strengths and limitations

This cross-sectional study is the first investigating adherence of AYA cancer survivors to the 2018 cancer prevention WCRF/AICR lifestyle recommendations. Another major strength is the big sample size, the long time since diagnosis, and the population-based character of the study. Moreover, this study focuses on the relation between lifestyle determinants on lifestyle as a whole.

This study also has some limitations. The use of a self-reported questionnaire to assess lifestyle habits might have led to an overestimation of MVPA levels, consumption of vegetables and fruit and underestimation of body weight, and alcohol consumption due to socially desirable answers [55, 56]. Misestimation of dietary habits could also have occurred due to the presence of closed answers in the questionnaire, and because we did not always had information on the amount of food consumed.

Self-reported body weight could have led to an underestimation of BMI, especially in overweight and obese survivors. Hence, it could be possible that survivors were classified in the healthy BMI group instead of the overweight/obese group, increasing the number of participants with a higher adherence to that recommendation, which might have influenced the final score. For physical activity, 98% of participants reported to adhere to the recommendations. This is a well-known problem of physical activity questionnaires, and we assume that this is a systematic error. Moreover, the questionnaire we used is a reduced version of the EPIC PAQ, and this could have led to further overestimation. The reduced number of questions might made it harder to estimate the intensity of PA, so that participants could have underestimated leisure activity and overestimated sports activities in terms of intensity of the effort. Moreover, since the study is conducted among a Dutch population that culturally has high levels of cycling, the total amount of time spent cycling and its intensity could have been overestimated [57]. Therefore, we decided to categorize our MVPA data in tertiles and expect that this may have reduced possible bias. Since monitoring devices are widely available, we recommend the use of objective physical activity measures (e.g., accelerometers in combination with a heart rate monitor) in future studies to confirm our findings [58].

In addition, insight from previous analyses that compared responders to non-responders in the same AYA cohort [59] showed that the responders are specific subgroups of AYA cancer survivors. Participation was more likely among females; AYAs with a higher socio-economic status (SES), those diagnosed over 10 years ago, individuals diagnosed with a central nervous system tumor, sarcoma, and a lymphoid malignancy, stage III, or those treated with systemic chemotherapy. Selection bias may limit the generalization of our findings, suggesting that individuals with unhealthy lifestyle, such as smokers and alcohol drinkers, might be less likely to participate, so lifestyle behaviors might be even worse in the general AYA population [60].

Moreover, due to the cross-sectional design of this observational study, it is not possible to assess causal relationships between determinants and WCRF/AICR recommendations adherence. As participants are long-term cancer survivors, it is unknown as to whether lifestyle habits changed after diagnosis. Future prospective longitudinal research is needed to study the level of adherence and its (eventual) change over time.

### Future suggestions and implications

Our findings suggest that (oncology) health care professionals and health promotion researchers should focus on promoting (maintenance of) adherence to a healthy lifestyle, especially in regard to body weight, fruit and vegetable intake, and alcohol consumption among AYA cancer survivors. Since long-term survivors report a poorer lifestyle than survivors shortly after diagnosis, health care providers should not only support behavior change immediately after diagnosis but also regularly check changes in behavior and continue lifestyle support in follow-up care during the whole cancer continuum. To discuss lifestyle topics with AYAs, health care professionals may use, for example, the anamnesis tool in the electronic medical record which was initiated and developed by the AYA Healthcare Network in the Netherlands [61]. This anamnesis includes questions about nutrition and sport activities to facilitate conversations between health care professionals and AYAs. Moreover, health care professionals should focus on mutual participation, raising awareness, providing necessary information, taking into account cultural interpretation and language differences, and open communication since these are critical strategies to empower AYA cancer survivors [62].

Many of the unhealthy lifestyle behaviors that AYA cancer survivors have could be addressed and ameliorated through interventions. Behavior change interventions on dietary habits, physical activity, body weight, and composition have been delivered to AYA cancer survivors, resulting in significant change in lifestyle [62–64]. Considering what has been reported in studies about behavioral change in AYA cancer survivors, it seems that the use of technology with emails, telephone calls, text messages, web-based electronic health (eHealth), games, or online quizzes may be a good way to convey messages on prevention and healthy lifestyle, since it is clear that AYA cancer survivors prefer remote-delivered interventions [14, 63]. In addition, it is important to monitor lifestyle interventions and behaviors: pedometer use has been shown not only to increase but also to monitor physical activity. Personal activity monitors (Zamzee, Fitbit, Jawbone UP, and Nike + Fuelband) have additional capabilities and can monitor sleep, adding inputted nutrition information and interfacing with social networks [65]. Apps (applications) for smartphones and tablets also have tracking abilities, and many of them are related to physical activity and nutrition. Moreover, some apps have social networking components which add engagement and have the potential to provide the social support that could motivate and reinforce change [66].

Both apps and websites would build a network of social support for AYA cancer survivors. In addition, posting by survivors would enhance the content (peer teaching) [67]. Those features encourage engagement and could contribute to the empowerment of AYAs, since they select to affiliate with those sharing common attitudes and interests, and these social interactions can have major influences on behavior.

Due to the limited knowledge of lifestyle in AYA cancer survivors, more studies are needed. It is necessary to encourage participation in programs about general health information and education and clinical trials immediately after diagnosis to investigate if and how AYAs could easily change their habits from that moment and if there are determinants over time that worsen their behaviors. More studies are also needed to test the efficacy of more recently introduced interventions and factors that could contribute to the adherence to health-promoting messages. This knowledge is essential for formulating specific lifestyle interventions and recommendations for AYA cancer survivors.

## Conclusion

Our study showed that adherence to the 2018 WCRF/AICR lifestyle recommendations is generally low to moderate in AYA cancer survivors, especially in regard to recommendations about body weight, and fruit, vegetables, and alcohol consumption. Men, people with a low education level, current smokers, and AYA cancer survivors diagnosed with germ cell tumors need more attention because they are less likely to have a healthy lifestyle compared to women, highly educated people, never-smokers, and AYA breast cancer survivors. More knowledge is needed on lifestyle and its determinants among AYA cancer survivors, focusing also on age-specific tools to stimulate and motivate AYA survivors to follow the recommendations to the best of their abilities.

## Supplementary Information

Below is the link to the electronic supplementary material.

Supplementary file1 (DOCX 491 KB)

## Data Availability

The data presented in this study are available on reasonable request from the corresponding author. The data are not publicly available due to privacy issues.
