# Supplementary material for: Adherence to the World Cancer Research Fund/American Institute for Cancer Research recommendations for cancer prevention in adolescent and young adult (AYA) cancer survivors: results from the SURVAYA study
Source: J Cancer Surviv. 2024 Jan 15;19(3):1028–42. doi: 10.1007/s11764-023-01529-4 (PMC12081526; doi:10.1007/s11764-023-01529-4)
Supplement: Supplementary file 1 — Supplementary file1 (DOCX 491 KB) [file 11764_2023_1529_MOESM1_ESM.docx]

**Supplementary file 1**

English translation of SURVAYA questions about lifestyle behaviours

|  | | Do you smoke? | | | | | | | | | | | | | | | | | |  |
| --- | --- | --- | --- | --- | --- | --- | --- | --- | --- | --- | --- | --- | --- | --- | --- | --- | --- | --- | --- | --- |
|  |  | | | | | | | | | | | | | | | | |  |  |  |
| ❒ | | No, I have never smoked | | | | | | | | | | | | | | |  |  |  |  |
|  |  | | | | | | | | | | | | | | | | |  |  |  |
| ❒ | | No, I don’t smoke, but I used to | | | | | | | | | | | | | | | | |  |  |
|  | | I have smoked for |  |  | year(s) | | | | | | | | | |  |  |  |  |  |  |
|  | | | | | | | | | | | | | | | | | | | | |
|  | | of which |  |  | year(s) before the cancer diagnosis | | | | | | | | | | |  |  |  |  |  |
|  |  | | | | | | | |  | | | |  |  | | | |  |  |  |
| ❒ | | Yes, I smoke for | | | |  | |  | | | year(s) | | | | | | |  |  |  |
|  | |  | | | |  |  | | |  | | | | | | | |  |  |  |
|  | | of which | | | |  | |  | | | year(s) before the cancer diagnosis | | | | | | |  |  |  |
|  | |  | | | |  |  | | |  | | | | | | | |  |  |  |
|  | | I smoke on average (over the past 12 months): | | | |  | |  | | | sigarettes per day | | | | | | |  |  |  |
|  | |  | | | |  | |  | | | |  | | | | | |  |  |  |
|  | |  | | | |  | |  | | | sigars per week | | | | | | |  |  |  |
|  | |  | | | |  | |  | | | |  | | | | | |  |  |  |
|  | |  | | | |  | |  | | | packet(s) of pipe tobacco (of 50 grams) per week | | | | | | |  |  |  |

| **Smoking, alcohol- and drugs use** |
| --- |

|  | | Do you drink alcohol? | | | | | | | | | | | | | | |  |
| --- | --- | --- | --- | --- | --- | --- | --- | --- | --- | --- | --- | --- | --- | --- | --- | --- | --- |
|  |  | | | | | | | | | | | | | |  |  |  |
| ❒ | | No, I don’t drink alcohol. | | | | | | | | | | | |  |  |  |  |
|  |  | | | | | | | | | | | | | |  |  |  |
| ❒ | | No, I don’t drink alcohol, but I used to | | | | | | | | | | | | | |  |  |
|  | | I drink alcohol for |  |  | year(s) | | | | | |  |  |  |  |  |  |  |
|  | | | | | | | | | | | | | | | | | |
|  | | of which |  |  | year(s) before the cancer diagnosis | | | | | | |  |  |  |  |  |  |
|  |  | | | | | |  | |  |  | | | | |  |  |  |
| ❒ | | Yes, I drink alcohol for | | | |  |  | year(s) | | | | | |  |  |  |  |
|  | |  | | | |  |  |  | | | | | |  |  |  |  |
|  | | of which | | | |  |  | year before the cancer diagnosis | | | | | |  |  |  |  |
|  | |  | | | |  |  |  | | | | | |  |  |  |  |
|  | | I drink on average (over the 12 months): | | | |  |  | glasses of beer per week | | | | | |  |  |  |  |
|  | |  | | | |  |  |  | | | | | |  |  |  |  |
|  | |  | | | |  |  | glasses of wine (or sherry/port) per week | | | | | |  |  |  |  |
|  | |  | | | |  |  |  | | | | | |  |  |  |  |
|  | |  | | | |  |  | glasses of hard liquor per week (for example: cognac, gin, whisky, liqueur, etc) | | | | | |  |  |  |  |
|  | | Have you ever used drugs? | | | | | | | | | | |  |  |  |  |  |
|  | |  | | | | | | | | | | |  |  |  |  |  |
| ❒ | | No | | | | | | | | | | |  |  |  |  |  |
| ❒ | | Yes | | | | | | | | | | |  |  |  |  |  |

|  | Below is a list of several type of drugs. Could you please verify for each type of drug whether you use it never, occasionally, annually, monthly, weekly or daily? |
| --- | --- |

|  |  | **Never** | **Occasionally** | | **Annually** | | **Monthly** | | **Weekly** | **Daily** | |  |  |
| --- | --- | --- | --- | --- | --- | --- | --- | --- | --- | --- | --- | --- | --- |
|  | Cannabis including hashish/marijuana, weed | ❒ | ❒ | | ❒ | | ❒ | | ❒ | ❒ | |  |  |
|  | Amphetamine | ❒ | ❒ | | ❒ | | ❒ | | ❒ | ❒ | |  |  |
|  | GHB | ❒ | ❒ | | ❒ | | ❒ | | ❒ | ❒ | |  |  |
|  | MDA, crystal, speed | ❒ | ❒ | | ❒ | | ❒ | | ❒ | ❒ | |  |  |
|  | Cocaine | ❒ | ❒ | | ❒ | | ❒ | | ❒ | ❒ | |  |  |
|  | Heroin | ❒ | ❒ | | ❒ | | ❒ | | ❒ | ❒ | |  |  |
|  | Ecstasy | ❒ | ❒ | | ❒ | | ❒ | | ❒ | ❒ | |  |  |
|  | Another type of drugs, namely: | | | | | | | | | | | | |
|  |  | | | | | | | | | | | |  |
|  |  | ❒ | ❒ | | ❒ | | ❒ | | ❒ | ❒ | |  |  |
|  |  |  |  | |  | |  | |  |  | |  |  |
|  |  |  |  | |  | |  | |  |  | |  |  |
|  |  | ❒ | ❒ | | ❒ | | ❒ | | ❒ | ❒ | |  |  |
|  |  |  |  | |  | |  | |  |  | |  |  |
|  |  |  |  | |  | |  | |  |  | |  |  |
|  |  | ❒ | ❒ | | ❒ | | ❒ | | ❒ | ❒ | |  |  |
|  |  |  | |  | |  | |  | | |  | |  |

| **Weight, height, body measurements, activities and nutrition** |
| --- |

|  | What is your height? *(If you don’t know exactly please give an estimate)* | | | | | |
| --- | --- | --- | --- | --- | --- | --- |
|  |  | | | | | |
|  | |  |  |  | cm |  |

|  | | What is your weight? *(If you don’t know exactly please give an estimate)* | | | | | | |
| --- | --- | --- | --- | --- | --- | --- | --- | --- |
|  |  | | | | | | |  |
|  | | |  |  |  | kg |  |  |

We would like you to measure the circumference of your waist using the measuring tape you received from us. You can do the measurement by yourself, but it’s most convenient if someone else can perform the measurement for you.

It’s best to measure while standing and ensure the measuring tape runs horizontally across both the belly and the back.


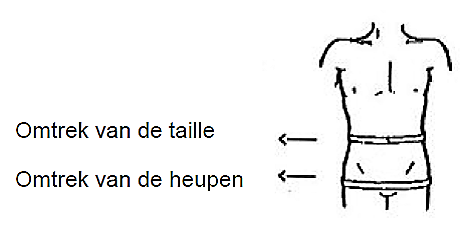


Refer to the picture to see exactly where you should measure.

Circumference of the waist

**Circumference of the taille**

Breathe in and out calmly and measure the circumference with the measuring tape exactly between the top of the hip bone and the bottom rib. Ensure the tape goes over your navel. Do not measure over thick clothing. It’s best to measure your bare belly.

|  | How many centimeter is the circumference of your taille? |  |  |  | cm |
| --- | --- | --- | --- | --- | --- |

|  | | Please could you indicate in the table below, how many hour you have spent on average on each of the following activities **during the past summer and winter**,   - *This refers to activities in addition to your possible (un)paid work .* - *If a type of activity not applicable, please enter ‘0’.* | | | | | | | | | | |  |  |
| --- | --- | --- | --- | --- | --- | --- | --- | --- | --- | --- | --- | --- | --- | --- |
|  | | | | |  | | | | | | | | | |
|  | | | | | **How many hours per week?** | | | | | |  |  |  |  |
| **Type physical activity during past summer and winter** | | | | | **Summer** | | | **Winter** | | |  |  |  |  |
|  | |  | | | | | | | | | |  |  |  |
| **Walking** (including walking to work, shopping and leisure time) | | | | |  |  | hours |  |  | hours |  |  |  |  |
|  | |  | | | | | | | | | |  |  |  |
| **Cycling** (including cycling to work, shopping and leisure time) | | | | |  |  | hours |  |  | hours |  |  |  |  |
|  | |  | | | | | | | | | |  |  |  |
| **Gardening** | | | | |  |  | hours |  |  | hours |  |  |  |  |
|  | |  | | | | | | | | | |  |  |  |
| **Household activities** (for example: washing, cleaning, cooking, caring for children) | | | | |  |  | hours |  |  | hours |  |  |  |  |

|  | Have you participated in sports **weekly, in the past year**? | |
| --- | --- | --- |
|  | |  |
| ❒ | No | |
| ❒ | Yes | |

| 11. | | If so, could you please indicate in the following table:   - 1. Type of sport   2. Whether this was/is in competitive context   3. Average number of hours per week - *Please only verify sports that you participated in weekly* | |
| --- | --- | --- | --- |
|  |  | |  |

|  | **Type of sport** | **In competitive context** | | **Average number of hours per week** | | | |  |
| --- | --- | --- | --- | --- | --- | --- | --- | --- |
|  |  | **Yes** | **No** |  |  |  |  |  |
| 1 |  | ❒ | ❒ |  |  | hours |  |  |
|  |  | | | | | | | |
| 2 |  | ❒ | ❒ |  |  | hours |  |  |
|  |  | | | | | | | |
| 3 |  | ❒ | ❒ |  |  | hours |  |  |
|  |  | | | | | | | |
| 4 |  | ❒ | ❒ |  |  | hours |  |  |
|  |  | | | | | | | |
| 5 |  | ❒ | ❒ |  |  | hours |  |  |
|  |  | | | | | | | |
| 6 |  | ❒ | ❒ |  |  | hours |  |  |

|  | How many days per week do you eat usually vegetables? |
| --- | --- |
|  |  |
| ❒ | Never or less than 1 day per week |
| ❒ | 1 day per week |
| ❒ | 2 days per week |
| ❒ | 3 days per week |
| ❒ | 4 days per week |
| ❒ | 5 days per week |
| ❒ | 6 days per week |
| ❒ | 7 days per week |

|  | On a day when you eat vegetables, how many serving spoons do you usually eat? |
| --- | --- |
|  |  |
| ❒ | 1 serving spoon per day |
| ❒ | 2 serving spoons per day |
| ❒ | 3 serving spoons per day |
| ❒ | 4 serving spoons per day |
| ❒ | 5 serving spoons per day |
| ❒ | 6 serving spoons per day |
| ❒ | 7 or more serving spoons per day |
| ❒ | Not applicable |

|  | How many days per week do you eat usually fruit? |
| --- | --- |
|  |  |
| ❒ | Never or less than 1 day per week |
| ❒ | 1 day per week |
| ❒ | 2 days per week |
| ❒ | 3 days per week |
| ❒ | 4 days per week |
| ❒ | 5 days per week |
| ❒ | 6 days per week |
| ❒ | 7 days per week |

|  | On a day when you eat fruit, how many pieces do you usually eat? |
| --- | --- |
|  |  |
| ❒ | 1 per day |
| ❒ | 2 per day |
| ❒ | 3 per day |
| ❒ | 4 per day |
| ❒ | 5 per day |
| ❒ | Not applicable |

|  | How many days per week do you usually eat cookies, cake, pie or chips? |
| --- | --- |
|  |  |
| ❒ | Never or less than 1 day per week |
| ❒ | 1 day per week |
| ❒ | 2 days per week |
| ❒ | 3 days per week |
| ❒ | 4 days per week |
| ❒ | 5 days per week |
| ❒ | 6 days per week |
| ❒ | 7 days per week |

|  | How many days per week do you usually eat red meat?  *Red meat is meat from mammals, such as cattle, pigs, goats, lambs, or horses. It also included meat in all forms, such as minced meat or chops.* |
| --- | --- |
|  |  |
| ❒ | Never or less than 1 day per week |
| ❒ | 1 day per week |
| ❒ | 2 days per week |
| ❒ | 3 days per week |
| ❒ | 4 days per week |
| ❒ | 5 days per week |
| ❒ | 6 days per week |
| ❒ | 7 days per week |

|  | How many days per week do you usually eat processed meat?  *Processed meat is meat that has been salted, smoked, dried, fermented or to which preservatives have been added. Examples are: deli meat, wurstel, smoked sausages.* |
| --- | --- |
|  |  |
| ❒ | Never or less than 1 day per week |
| ❒ | 1 day per week |
| ❒ | 2 days per week |
| ❒ | 3 days per week |
| ❒ | 4 days per week |
| ❒ | 5 days per week |
| ❒ | 6 days per week |
| ❒ | 7 days per week |

|  | How many days a week do you drink sugary soft drinks (not diet), sugary lemonade, or sport drinks? |
| --- | --- |
|  |  |
| ❒ | Never or less than 1 day per week |
| ❒ | 1 day per week |
| ❒ | 2 days per week |
| ❒ | 3 days per week |
| ❒ | 4 days per week |
| ❒ | 5 days per week |
| ❒ | 6 days per week |
| ❒ | 7 days per week |

|  | On a day when you drink sugary soft drinks (not diet), sugary lemonade, or sportdrinks, how many do you usually drink? |
| --- | --- |
|  |  |
| ❒ | 1 glass per day |
| ❒ | 2 glasses per day |
| ❒ | 3 glasses per day |
| ❒ | 4 glasses per day |
| ❒ | 5 glasses per day |
| ❒ | 6 glasses per day |
| ❒ | 7 or more glasses per day |
| ❒ | Not applicable |

|  | How many days a week do you usually eat fast food (hamburgers, fries, pizzas, milkshakes etc)? |
| --- | --- |
|  |  |
| ❒ | Never or less than 1 day per week |
| ❒ | 1 day per week |
| ❒ | 2 days per week |
| ❒ | 3 days per week |
| ❒ | 4 days per week |
| ❒ | 5 days per week |
| ❒ | 6 days per week |
| ❒ | 7 days per week |

**Supplementary file 2**.

**Fig. 1a – 1f.** Distributions of BMI, waist circumference and consumption of Vegetables, Fruit, Sweetened drinks and Alcoholic drinks among participants of the study, by WCRF/AICR cancer prevention recommendations cut-offs.

|  |  |
| --- | --- |
|  |  |
|  |  |

Abbreviations: M, men; W, women.
